# Cellulose Nanofiber Biotemplated Palladium Composite Aerogels

**DOI:** 10.3390/molecules23061405

**Published:** 2018-06-09

**Authors:** Fred J. Burpo, Alexander N. Mitropoulos, Enoch A. Nagelli, Jesse L. Palmer, Lauren A. Morris, Madeline Y. Ryu, J. Kenneth Wickiser

**Affiliations:** 1 Department of Chemistry and Life Science, United States Military Academy, West Point, NY 10996, USA; alex.mitropoulos@usma.edu (A.N.M.); enoch.nagelli@usma.edu (E.A.N.); jesse.palmer@usma.edu (J.L.P.); madeline.ryu@usma.edu (M.Y.R.); john.wickiser@usma.edu (J.K.W.); 2 Department of Mathematical Sciences, United States Military Academy, West Point, NY 10996, USA; 3 Armament Research, Development and Engineering Center, U.S. Army RDECOM-ARDEC, Picatinny Arsenal, NJ 07806, USA; lauren.a.morris17.civ@mail.mil

**Keywords:** aerogels, palladium, porous, nanomaterials, catalysis

## Abstract

Noble metal aerogels offer a wide range of catalytic applications due to their high surface area and tunable porosity. Control over monolith shape, pore size, and nanofiber diameter is desired in order to optimize electronic conductivity and mechanical integrity for device applications. However, common aerogel synthesis techniques such as solvent mediated aggregation, linker molecules, sol–gel, hydrothermal, and carbothermal reduction are limited when using noble metal salts. Here, we present the synthesis of palladium aerogels using carboxymethyl cellulose nanofiber (CNF) biotemplates that provide control over aerogel shape, pore size, and conductivity. Biotemplate hydrogels were formed via covalent cross linking using 1-ethyl-3-(3-dimethylaminopropyl) carbodiimide hydrochloride (EDC) with a diamine linker between carboxymethylated cellulose nanofibers. Biotemplate CNF hydrogels were equilibrated in precursor palladium salt solutions, reduced with sodium borohydride, and rinsed with water followed by ethanol dehydration, and supercritical drying to produce freestanding aerogels. Scanning electron microscopy indicated three-dimensional nanowire structures, and X-ray diffractometry confirmed palladium and palladium hydride phases. Gas adsorption, impedance spectroscopy, and cyclic voltammetry were correlated to determine aerogel surface area. These self-supporting CNF-palladium aerogels demonstrate a simple synthesis scheme to control porosity, electrical conductivity, and mechanical robustness for catalytic, sensing, and energy applications.

## 1. Introduction

Natural biomaterials display several examples of forming controlled structures with dimensions observed at the submicron and nanometer scales [1]. Recently, generating feature sizes at these length scales has been of interest using biopolymers such as proteins, carbohydrates, and nucleic acids because these molecules’ innate structure can be controlled at nanometer dimensions in bulk materials due to natural self-assembly [1,2]. However, even though nature uses these molecules at nano-dimensions, engineering new structures with the same materials at these length scales is challenging as the current technology in micro and nanofabrication, such as lithography or e-beam writing are not easily compatible with biomaterials [2,3]. Chemical vapor deposition (CVD) has been shown to produce exact replicas of biological specimens as templates leave the underlying structure unharmed [4]. However, CVD requires expensive equipment and control to accurately template biostructures. Additionally, current micro- and nanofabrication methods generally produce thin-film structures and do not allow for easy formation of bulk nanostructured three-dimensional networks [2,5]. A synthesis route for assembling bulk nanostructures relies on the natural self-assembly of biomaterials such as cellulose, which use intrinsic molecular interactions to produce hierarchical nano-networks, while offering flexibility and biocompatibility, making these materials ideal to be used as templates [6,7].

Cellulose is the most abundant natural polymer and primary structural component found in cell walls and the components of wood known for its strength and flexibility [8,9,10,11]. Made from a linear chain of β(1→4) d-glucose molecules, cellulose functions as an energy storage and structural molecule found primarily in the cell wall of many plants and bacteria [8]. However, the properties of cellulose can be engineered by processing individual cellulose molecules into nanocrystalline structures to generate cellulose nanofibers (CNFs), a form of cellulose where several molecules assemble together to form fibers with a high aspect ratio approximately 20 nanometers in diameter and hundreds of nanometers to several microns in length [12,13,14,15,16,17]. Additionally, to improve the function and capacity of CNFs, cellulose can be modified with carboxymethyl groups to enhance gelation and ionic conjugation giving CNFs additional properties [18,19,20]. 

Nanocellulose and CNFs have interested researchers over the last few decades because they offer the strength and flexibility of cellulose with dimensions that can be used to produce films and hydrogels that are stronger than cellulose in its innate form [21,22,23,24]. Additionally, CNFs are water soluble and renewable materials that do not require harsh organic solvents or drastic changes in pH to process [12,25]. The hydroxyl group found in cellulose can be functionalized with a carboxymethyl group facilitating CNFs to form freestanding gels with high porosity and large surface area [26,27,28,29]. Therefore, optimizing the concentration can generate robust hydrogels with high porosity, and allow CNFs to act as a biotemplate for the electrochemical deposition of metals. In their hydrated state, carboxymethyl CNF hydrogels can electrostatically bind metal cations that attach to negatively charged functional groups adding increased flexibility to this material platform [6,7,30,31,32,33,34].

Noble metal salts easily form nanoparticles with common reducing agents in numerous synthesis methods. Nobel metal aerogels have been formed via sol-gel, solvent mediated aggregation, select alloy etching, linker molecules, and direct reduction [35,36,37,38,39]. Additionally, three-dimensional nanostructures have been achieved using biotemplating with noble metal salts binding to oppositely charged template surfaces. In this manner, charge distribution along biopolymer chains allows the metal salts to be deposited where the polymer acts as a structural template making a composite material [40,41,42]. Upon reduction with reducing agents such as sodium borohydride, biotemplates are metalized to make them conductive [39,43]. CNF hydrogels incubated in noble metals salts and chemically reduced can produce discrete templated nanoparticles along the CNF surface with the flexibility and mechanical properties of cellulose [31,32]. Maintaining the high porosity of CNF hydrogel biotemplated metal structure can be accomplished by supercritically drying with CO_2_ generating composite CNF aerogels [26,44,45]. Through this process, an insulating biopolymer becomes conductive via metallization providing unique opportunities to electrically, chemically, or optically functionalize these materials.

Here, we demonstrate a method to use covalently cross-linked carboxymethylated cellulose nanofibers as a nanostructured template network to form three-dimensional CNF-palladium composite aerogels. Equilibration of CNF hydrogels in palladium salt solutions allows for electrostatic binding of palladium ions onto the carboxylic acid functional groups on the biotemplate cellulose nanofibers. The high surface area and porosity associated with the CNF hydrogel confers similar properties to the metalized aerogel. Reduced with sodium borohydride and then supercritically dried, these CNF aerogels combine the unique physical and mechanical properties of cellulose with the conductivity of noble metals giving them potential as advanced energy, catalysis, and sensing platforms.

## 2. Materials and Methods

### 2.1. Hydrogel Preparation

Carboxymethyl cellulose nanofibers (CNF) (University of Maine, Process Development Center, Orono, ME, USA) with a nominal 300 nm length and carboxymethyl concentration of 1.2 mmol/g were used to prepare hydrogels. CNF hydrogels were prepared from 3 % (*w*/*w*) CNF solution in deionized water. In addition, 0.25 mL of the CNF solution was centrifuged for 20 min at 21,000 × *g* that increased the CNF concentration to 3.8% (*w*/*w*). Solutions presented a translucent white color after centrifugation. CNF hydrogels were crosslinked in 0.5 M 1-ethyl-3-(3-dimethylaminopropyl) carbodiimide hydrochloride (EDC) (Sigma Aldrich, Allentown, PA, USA) with 0.1 M MES buffer (Sigma Aldrich, Allentown, PA, USA), 0.25 M ethylenediamine (EDA) (Sigma Aldrich, USA) at pH 4.5 for 48 h. Samples were rinsed in deionized water for 24 h before equilibrating the CNF hydrogels with 1, 10, 50, 100, 500, and 1000 mM concentrations of either sodium tetrachloropalladate (II), or tetraamminepalladium (II) chloride (Sigma Aldrich, Allentown, PA, USA) for 24 h. Samples were reduced in 2.0 M sodium borohydride, NaBH_4_, for 24 h followed by 0.5 M NaBH_4_ (Sigma Aldrich, Allentown, PA, USA) for 12 h. Palladium composite gels were rinsed in deionized water for 24 h. To maintain the palladium coated nanofibrillar hydrogel network, samples were then dehydrated in a series of ethanol rinses at concentrations of 25, 50, 75, and 100% (*v*/*v*) in deionized water for 30 min each and then supercritically dried in CO_2_ using a Leica EM CPD300 Automated Critical Point Dryer (Leica Biosystems, Buffalo Grove, IL, USA) with a set point of 35 °C and 1200 psi.

### 2.2. Scanning Electron Microscopy

Scanning electron microscopy (SEM) was used to evaluate scaffold morphology. All micrographs were taken with a FEI Helios 600 scanning electron microscope (ThermoFisher Scientific, Hillsboro, OR, USA). Samples were coated with gold for 30 s using a Desk V Denton sputter coater (Denton Vacuum, Moorestown, NJ, USA).

### 2.3. Thermal Gravimetric Analysis (TGA)

Thermal gravimetric analysis (TGA) was performed on a Thermal Instruments Q-500 (Thermal Instruments, New Castle, DE, USA) in a ramp state with a temperature rate of 10 °C/min from ambient to 700 °C. Samples were in nitrogen gas with a flow rate of 60 mL/min.

### 2.4. X-Ray Diffractometry

X-ray diffractometry (XRD) was performed with a PANalytical Emperean diffractometer (Malvern PANalytical, Almelo, the Netherlands). XRD scans for diffraction angles (2 θ) from 5 ° to 90 ° were performed at 45 kV and 40 mA with Cu K_α_ radiation (1.54060 Å), a 2 θ step size of 0.0130 °, and 20 s per step. XRD spectra analysis was performed with High Score Plus software (Version 4.6, Malvern PANalytical, Almelo, the Netherlands).

### 2.5. BET Analysis

Adsorption–desorption measurements were performed according to IUPAC standards [46] using a Quantachrome NOVA 4000e (Quantachrome Instruments, Boynton Beach, FL, USA) surface area and pore size analyzer with nitrogen (−196 °C) as the test gas. All samples were vacuum degassed at room temperature for 24 h prior to measurement. Brunauer–Emmett–Teller (BET) analysis [47] was used to determine the specific surface area from gas adsorption. Pore-size distributions for each sample were calculated using the Barrett–Joyner–Halenda (BJH) model [48] applied to volumetric desorption isotherms. All calculations were performed using Quantachrome’s NovaWin software (Version 11.04, Quantachrome Instruments, Boynton Beach, FL, USA).

### 2.6. Electrochemical Characterization

A Bio-Logic VMP-3 potentiostat was used to perform electrochemical impedance spectroscopy (EIS) and cyclic voltammetry (CV) (BioLogic Science Instruments, Knoxville, TN, USA). A three electrode cell was used with an Ag/AgCl reference electrode, a 1 mm platinum wire counter-electrode, and a lacquer coated 1 mm platinum wire with exposed tip in contact with samples. In addition, 0.5 M H_2_SO_4_ electrolyte was used for EIS and CV. EIS was performed at open circuit voltage with a frequency range of 1 MHz to 1 mHz with a 10 mV sine wave. CV was performed in a voltage range of −0.2 to 1.2 V (vs. Ag/AgCl) with scan rates of 1, 5, 10, 25, 50, 75, and 100 mV/s.

## 3. Results and Discussion

### 3.1. Aerogel Synthesis

**Figure 1:**
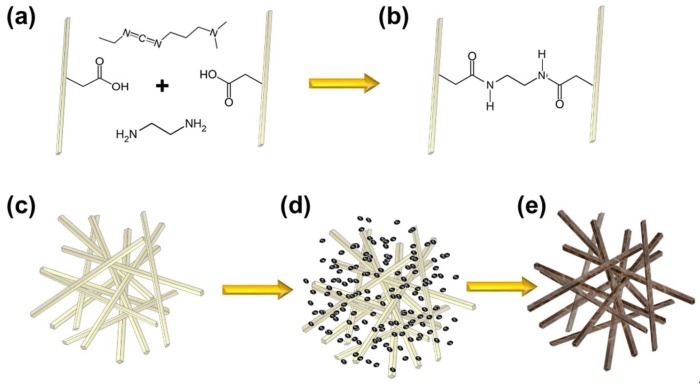
Aerogel synthesis scheme. (**a**) cross linking carboxymethyl cellulose nanofibers (CNF) with EDC and ethylenediamine as a linker molecule; (**b**),(**c**) cross-linked carboxymethyl cellulose nanofibers; (**d**) CNF hydrogel equilibrated with palladium salt solution; (**e**) CNF biotemplated palladium composite aerogel after reduction with NaBH_4_, rinsing, solvent exchange with ethanol, and supercritical drying.

Figure 1 depicts the synthesis scheme for cellulose nanofiber (CNF) palladium composite aerogels. Commercially available carboxymethylated CNFs were used in order to control the biotemplate surface charge by tuning the pH, and consequently the deprotonation of the carboxyl groups. At pH values lower than the carboxyl groups’ pKa of approximately 4.34, the negatively charged carboxylate groups serve as electrostatic binding sites for positively charged metal cations [49]. CNF hydrogels were prepared by centrifuging CNF solutions to ensure overlap of the cellulose nanofibers forming a physically entangled gel. Figure 1a depicts 1-ethyl-3-(3-dimethylaminopropyl) carbodiimide hydrochloride (EDC) with an ethylenediamine linker diffused into the compacted CNF gel. EDC molecules form an amide bond between one of the carboxyl groups on the cellulose nanofibers and one amine group on the ethylenediamine molecule. The second amine on ethylenediamine is then available to form a second amide linkage with an adjacent CNF through the coupling with another EDC molecule. In this manner, the carboxymethyl cellulose nanofibers are covalently linked to improve structural stability as shown in Figure 1b,c [50]. The resulting covalent hydrogels are then equilibrated in palladium salts solutions (Figure 1d), and then immersed in NaBH_4_ solution to reduce palladium ions within the gel. The metalized CNF-Pd composite gel is rinsed, solvent exchanged with ethanol, and supercritically dried to yield a CNF-Pd composite aerogel depicted in Figure 1e. 

**Figure 2:**
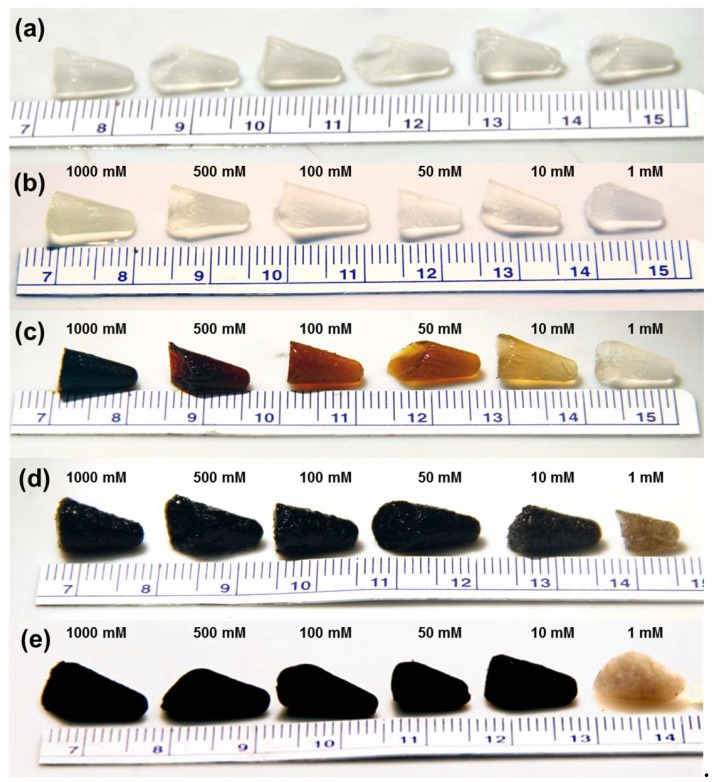
Aerogel synthesis process photos. (**a**) cross linked carboxymethyl cellulose nanofiber hydrogels with EDC and ethylenediamine as a linker molecule. CNF hydrogels equilibrated with palladium salt solutions of 1, 10, 50, 100, 500, and 1000 mM for (**b**) Pd(NH_3_)_4_Cl_2_, and (**c**) Na_2_PdCl_4_; (**d**) CNF biotemplated palladium aerogel after reduction with NaBH_4_; (**e**) CNF-Pd composite aerogels after rinsing, solvent exchange with ethanol and supercritical drying.

Figure 2 shows photo images of the synthesis scheme depicted in Figure 1 from covalent hydrogel formation through supercritical drying. Cellulose hydrogels shown in Figure 2a were crosslinked in microfuge tubes after compaction with centrifugation and then rinsed in deinionized water. After rinsing, CNF hydrogels were equilibrated in palladium salt solution with concentrations of 1, 10, 50, 100, 500, and 1000 mM. Both Pd(NH_3_)_4_Cl_2_ (Figure 2b) and Na_2_PdCl_4_ (Figure 2c) salt solutions, with corresponding complex ions of [Pd(NH_3_)_4_]^2+^ and [PdCl_4_]^2−^ were used with the synthesis scheme in Figure 1. The negatively charged [PdCl_4_]^2-^ ion exhibits a brown color correlating to the equilibrated ion concentration, whereas the pale yellow color of [Pd(NH_3_)_4_]^2+^ exhibits a less pronounced color correlation with concentration. Despite the osmotic pressures experienced by the CNF hydrogels solvated in water and exposed to increasing concentrations of palladium salt solutions up to 1000 mM, no gel swelling or de-swelling was observed for gels covalently cross-linked with EDC. In the absence of EDC mediated covalent cross-linking, physically entangled CNF hydrogels swelled and disaggregated in the presence of salt solutions, indicating increased gel stability with covalent cross-linking. 

Hydrogels equilibrated in palladium solution were then reduced in 2.0 M NaBH_4_ to form CNF-Pd composite gels. Exposure to such a high reducing agent concentration resulted in violent hydrogen gas evolution as a by-product of the electrochemical reduction; however, Figure 2d demonstrates that gels remained intact after reduction. Gels prepared with palladium solutions equal or greater to 50 mM were dark black, while gels using 10 mM and 1mM palladium solutions were slightly translucent. Gels were then rinsed in deionized water for at least 24 h to ensure that all excess reducing agent solution was removed from the CNF-Pd composite gel before solvent exchange to dehydrate the sample with ethanol. The dehydrated gels were then supercritically dried and are shown in Figure 2e. Reduced gels before and after supercritical drying presented an equivalent macroscopic appearance for gels prepared with both [PdCl_4_]^2−^ and [Pd(NH_3_)_4_]^2+^ solutions.

### 3.2. Scanning Electron Microscopy 

**Figure 3:**
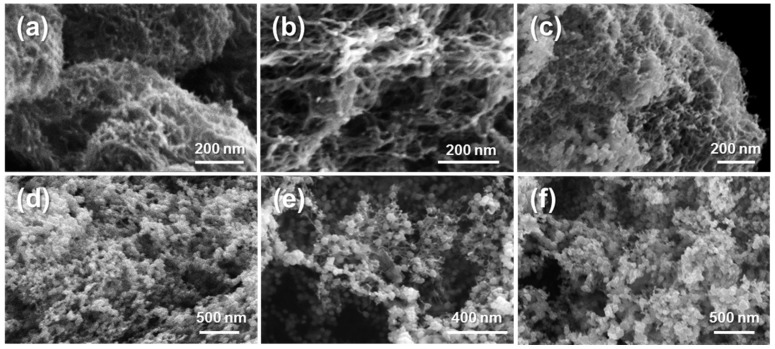
Scanning electron microscopy images of CNF-Pd composite aerogels prepared from Pd(NH_3_)_4_Cl_2_ concentrations of (**a**) 1 mM; (**b**) 10 mM; (**c**) 50 mM; (**d**) 100 mM; (**e**) 500 mM; and (**f**) 1000 mM.

Figure 3 shows scanning electron micrographs of CNF-Pd composite aerogels prepared using 1, 10, 50, 100, 500, and 1000 mM Pd(NH_3_)_4_Cl_2_. For aerogels prepared with 1 mM palladium solutions (Figure 3a), resulting aerogels present interconnected fibrillar elements with an average diameter of 12.6 ± 2.2 nm and pore sizes of 32.4 ± 13.3 nm, and a diameter of 12.4 ± 2.0 nm and pore size of 32.2 ± 10.4 nm for 10 mM samples (Figure 3b). For aerogels prepared with palladium concentrations of 50 mM and above (Figure 3c–f), the nanostructure changes compared to the lower concentrations to exhibit more pronounced interconnected nanoparticles. For the 50, 100, 500, and 1000 mM palladium synthesis concentrations, the average nanoparticle diameters are: 19.5 ± 5.0 nm, 41.9 ± 10.0 nm, 45.6 ± 14.6 nm, and 59.0 ± 16.4 nm. The nanoparticle size generally correlates with the equilibrated palladium precursor solution.

The observed nanostructure is similar to palladium and platinum structures synthesized using a direct reduction method using the same reagents at 100 mM concentrations in the absence of biotemplate [39]. Whereas the previously reported direct reduction method relies on hydrogen gas evolution for the coalescence of nanoparticles to form an aerogel monolith, it lacks macroscopic shape control compared to the method presented here with a defined shape biotemplate. The presence of the CNF aerogel not only guides the macroscopic monolith shape, but also serves as an anchor for nanoparticle attachment. We propose that the formation of the nanostructures occurs via palladium cations, [Pd(NH_3_)_4_]^2+^, electrostatically bound to deprotonated carboxyl (COO^−^) groups reduced to form initial nanoparticles along the surface of the cellulose nanofibers, allowing for fusion of nanoparticles formed within the hydrogel pores through surface free energy minimization. In the absence of crosslinking, ionic gels formed by centrifuging CNF solutions in the presence of palladium salt solutions, and reduced with NaBH_4_ resulted in nanofoams that did not maintain their macroscopic shape (Figure S1).

### 3.3. X-Ray Diffractometry

**Figure 4:**
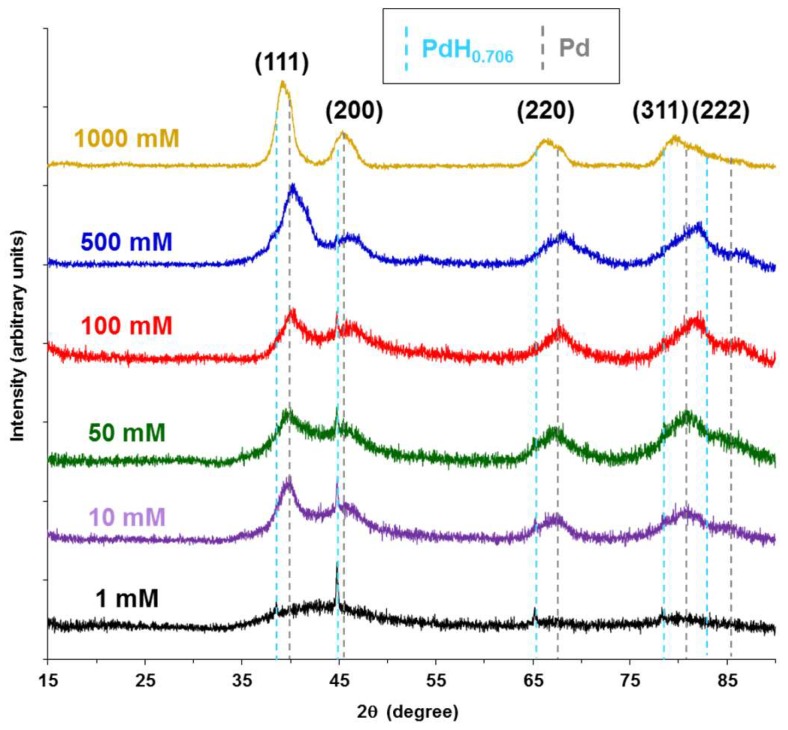
X-ray diffraction spectra for CNF-Pd composite aerogels synthesized from Pd(NH_3_)_4_Cl_2_ salt solution concentrations of 1 mM, 10 mM, 50 mM, 100 mM, 500 mM, and 1000 mM. JCPDS reference 00-018-0951 palladium hydride peak positions are indicated with a light blue dashed line, and dashed gray lines for 01-087-0643 palladium peak positions.

Figure 4 shows the X-ray diffraction spectra for CNF-Pd composite aerogels synthesized with 1, 10, 50, 100, 500, and 1000 mM Pd(NH_3_)_4_Cl_2_. For the lower synthesis concentrations and consequent lower metal to organic mass ratio, the XRD signal-to-noise ratio is low, but increases for aerogels prepared with 500 mM and 1000 mM palladium salt solutions. Spectra peak positions for all synthesis concentrations are broad indicating small crystallite sizes, and did not index to a single phase of palladium. For aerogels prepared with palladium solutions 100 mM and below, the presence of PdH_0.706_ palladium hydride peaks, indexed to Joint Committee on Powder Diffraction Standards (JCPDS) reference number 00-018-0951, were distinctly seen at 38.7°, 45.0°, 65.6°, and 78.9° for the (111), (200), (220), and (311) Miller indices, respectively. At room temperature, palladium hydride may exist in an α and β phase, where the hydrogen to palladium ratio for α phase is 0.03, and approximately 0.6 for the β phase [51]. The presence of the palladium hydride phase suggests that during the electrochemical reduction with NaBH_4_ hydrogen gas is entrained within the palladium nanoparticles [51]. With a 2.0 M NaBH_4_ reducing agent, hydrogen gas evolution within the CNF hydrogel pores is thought to generate sufficient gas pressure to drive the formation of the palladium hydride phase. The palladium phase in the aerogels was indexed to JCPDS reference 01-087-0643. Like palladium hydride, the palladium phase has a cubic crystal system, and a Fm-3m space group. As the palladium synthesis concentrations increase from 1 mM to 1000 mM, the distinct palladium hydride peaks become convoluted with the palladium phase peaks, such that they are not distinguishable at 1000 mM. Peak broadening decreases as the palladium synthesis concentrations increase corresponding to the average fiber diameters determined from SEM images in Figure 3. At 1 mM, the (111) and (200) peaks are combined and very broad, resolving as distinguishable peaks at synthesis concentrations above 100 mM. Similar XRD spectra were achieved for aerogels prepared using Na_2_PdCl_4_ (Figure S2).

### 3.4. Thermalgravimetric Analysis (TGA)

**Figure 5:**
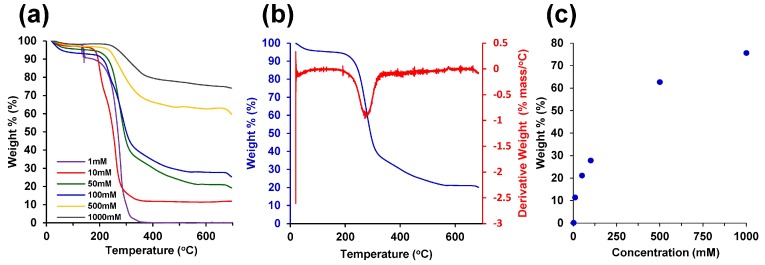
Thermogravimetric analysis (TGA). (**a**) TGA of aerogels synthesized with Pd(NH_3_)_4_Cl_2_ salt solutions; (**b**) TGA of 50 mM Pd(NH_3_)_4_Cl_2_ sample from (**a**) with differential thermal analysis (DTA); (**c**) palladium sample mass at 600 °C from (**a**) for the varying palladium concentrations.

To characterize the ratio of palladium and CNFs in the composite aerogels synthesized using Pd(NH_3_)_4_Cl_2_, thermogravimetric analysis (TGA) was performed with results shown in Figure 5. TGA revealed higher weight percentages of palladium in CNF-Pd composite aerogels synthesized from hydrogels equilibrated with higher concentration palladium solutions. Cellulose decomposes beyond 300 °C, as observed in the change in curve shape analyzed by the derivative weight shown in Figure 5a,b [52]. After complete decomposition of the CNF network, the remaining mass at 600 °C is the reduced palladium (Figure 5c). Similar results were achieved for aerogels prepared using Na_2_PdCl_4_ (Figure S3). The resulting metal to CNF mass ratios range from 0.002 to 3.1, and 0.07% to 75.5% for 1 mM to 1000 mM, respectively (Figure 5c).

### 3.5. Nitrogen Gas Adsorption

**Figure 6:**
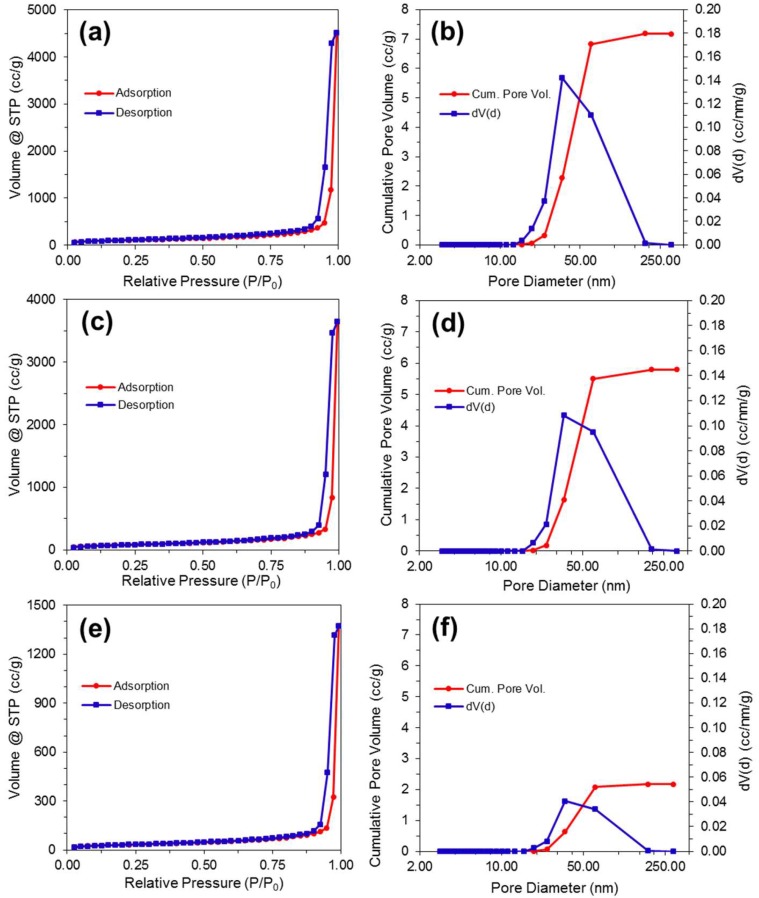
BET analysis. Nitrogen adsorption–desorption isotherms, and pore size distribution with cumulative pore volume for aerogels synthesized with palladium Pd(NH_3_)_4_Cl_2_ salt solutions of (**a**,**b**) 0 mM; (**c**,**d**) 100 mM; (**e**,**f**) 1000 mM.

Nitrogen gas adsorption isotherms were generated for CNF-Pd aerogels prepared with 0 mM, 100 mM, and 1000 mM Pd(NH_3_)_4_Cl_2_. The BET specific surface area of the 0 mM, 100 mM, and 1000 mM samples was 582, 456, and 171 m^2^/g, respectively, indicating that the specific surface area decreases in aerogels equilibrated with increasing concentration of palladium salt. The physisorption data shown in Figure 6 illustrates type IV adsorption–desorption isotherms in accordance with the IUPAC classification standards, revealing both mesoporous (2–50 nm in diameter) and macroporous (> 50 nm in diameter) structures in the CNF and CNF-Pd composite aerogel samples. The nitrogen adsorption quantity rises sharply for all samples at high relative pressures and no limiting adsorption is observed, consistent with the presence of both mesopores and macropores [47]. At relative pressure P/P_0_ = 0.995, the maximum volume adsorbed for the 0 mM, 100 mM, and 1000 mM samples was 4512, 3653, and 1372 cm^3^/g, respectively. H3 type hysteresis is observed in all samples, characteristic of capillary condensation in the mesopores. The hysteresis closes at higher relative pressures for increasing Pd concentrations as compared to the 0 mM sample, indicating that the smaller mesopores (< 30 nm) are eliminated by the increasing addition of the Pd phase; this result is consistent with the Barrett–Joyner–Halenda (BJH) pore size analysis, which shows a decreasing frequency of mesopores with the increasing addition of Pd [48]. The increasing blockage of mesopores with higher concentrations of palladium salt is evident in the pore volume distribution curves and is consistent with the observed reduction in specific surface area. BJH analysis of the desorption curve shows that the cumulative pore volume for the 0 mM, 100 mM, and 1000 mM samples was 7.171, 5.801, and 2.171 cm^3^/g, respectively. For CNF hydrogels with fixed pore sizes via covalent crosslinking, the reduction of higher Pd ion concentration results in increased metal content within the pores, and consequently the decreased cumulative pore volume observed with BJH analysis.

Sample porosities were calculated for the 0 mM, 100 mM, and 1000 mM aerogel samples from Equation (1):

% Porosity = (V_pores_/V_sample_) × 100%, (1)

with V_pores_ and V_sample_ as total pore and bulk sample volumes, respectively. The average bulk sample volumes of the 0 mM, 100 mM and 1000 mM samples were determined by measuring the volume and weight of three of each of the samples and calculating the average value. The resulting sample specific volumes for the 0 mM, 100 mM, and 1000 mM samples were 7.37 cm^3^/g, 6.10 cm^3^/g, and 2.40 cm^3^/g, respectively. The corresponding pore volumes were taken from the cumulative pore volume from the gas adsorption data. The resulting porosities for the 0 mM, 100 mM, and 1000 mM samples were 97.3%, 95.0%, and 90.4%.

### 3.6. Electrochemical Characterization

**Figure 7:**
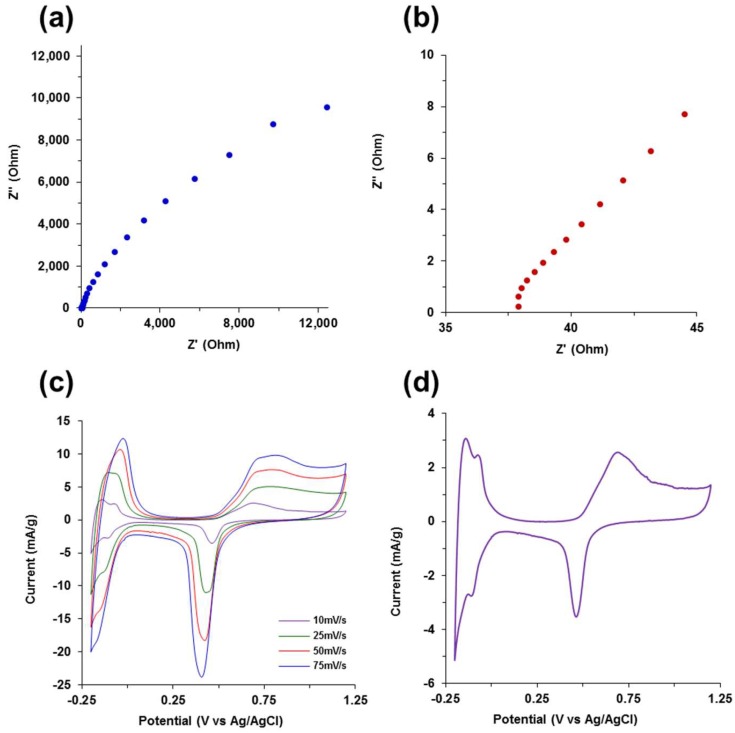
Electrochemical characterization in 0.5 M H_2_SO_4_ of CNF-Pd aerogels prepared from 1000 mM Pd(NH_3_)_4_Cl_2_. (**a**) electrochemical impedance spectroscopy (EIS) with a 10 mV sine wave was used across frequencies from 140 kHz to 15 mHz; (**b**) high frequency spectra from 140 kHz to 1.3 kHz from (**a**); (**c**) cyclic voltammetry (CV) at scan rates of 10, 25, 50, and 75 mV/s; (**d**) CV scan at 10 mV/s from (**c**).

The electrochemical analysis using CV and EIS techniques is shown in Figure 7. The Nyquist plot from 140 kHz to 15 mHz depicted in Figure 7a,b shows an incomplete semi-circle in the high frequency region indicating low resistance to charge transfer and double layer capacitance in the electrode/electrolyte interface. During the transition from high scan frequencies to low frequencies, there is a deviation from ideal capacitive behavior as the slope of the line starts to decrease toward the real axis due to non-ideal double layer capacitance from the non-uniform current distribution through the aerogel network, likely due to the composite mixture of metal and organic phases in the aerogels. The specific capacitance from the EIS plot at 15 mHz in Figure 7a was determined to be 40.6 mF/g using the relation:

C_sp_ = 1/(2πZ′′*f*m), (2)

with *f* as the frequency, Z" as the impedance imaginary component, and m as the sample palladium mass. A transmission line equivalent circuit model developed for porous noble metal aerogels was fit to the Nyquist plot in Figure 7a [39]. The transmission line model and resultant Nyquist plot model fit in Figure S5 are based on a modified Randles circuit consisting of parallel and serially connected resistors, constant phase elements or a capacitors, and restrictive diffusion elements through the hierarchical porous biotemplate aerogel network [53,54,55].

The CV scans at 10, 25, 50, and 75 mV/s in 0.5 M H_2_SO_4_ electrolyte shown in Figure 7c exhibit characteristic hydrogen adsorption–desorption, and the redox peaks associated with palladium. The CV scan at 10 mV/s in Figure 7d clearly shows the defined current response resulting from hydrogen adsorption and desorption peaks between −0.2 V and 0 V (vs. Ag/AgCl), along with palladium oxidation–reduction peaks more positive than +0.5 V. The predominant capacitive region between +0.1 and +0.3 V (vs. Ag/AgCl) yields a specific capacitance for the metal content of the aerogel of 34.7 mF/g, which is similar to the value determined from EIS. 

## 4. Conclusions

We have shown here that covalently cross-linked cellulose nanofiber hydrogels serve as a robust biotemplate to synthesize porous, high surface area, electrochemically active CNF-palladium composite aerogels. The CNF hydrogel biotemplate maintains its monolithic shape during all synthesis steps, and the carboxymethyl groups provide electrostatic binding sites for palladium cations and subsequent reduction of nanoparticles. This work demonstrates the ability to control the palladium metal content within a CNF hydrogel, and consequently control the performance characteristics of the material. CNF-palladium composite aerogels demonstrate a synthesis route to potentially use a variety of biological template hydrogels with pH tunable surface charge with other noble and transition metals compatible with aqueous solution reduction chemistries for a wide range of energy storage, catalysis, and sensing applications.

## Supplementary Materials

The following are available online, Figure S1: SEM images of nanofoams prepared from CNF ionic hydrogels, Figure S2: X-ray diffraction spectra for CNF-Pd aerogels synthesized from Na_2_PdCl_4_, Figure S3: TGA of aerogels synthesized with Na_2_PdCl_4_, Figure S4: Nitrogen gas adsorption–desorption overlay, and Figure S5: EIS Fitting.
