# Supplementary material for: Cellulose Nanofiber Biotemplated Palladium Composite Aerogels
Source: Molecules. 2018 Jun 9;23(6):1405. doi: 10.3390/molecules23061405 (PMC6100374; doi:10.3390/molecules23061405)
Supplement: Supplementary file 1 [file molecules-23-01405-s001.zip › molecules-308990-supplementary.pptx]

## Slide 1
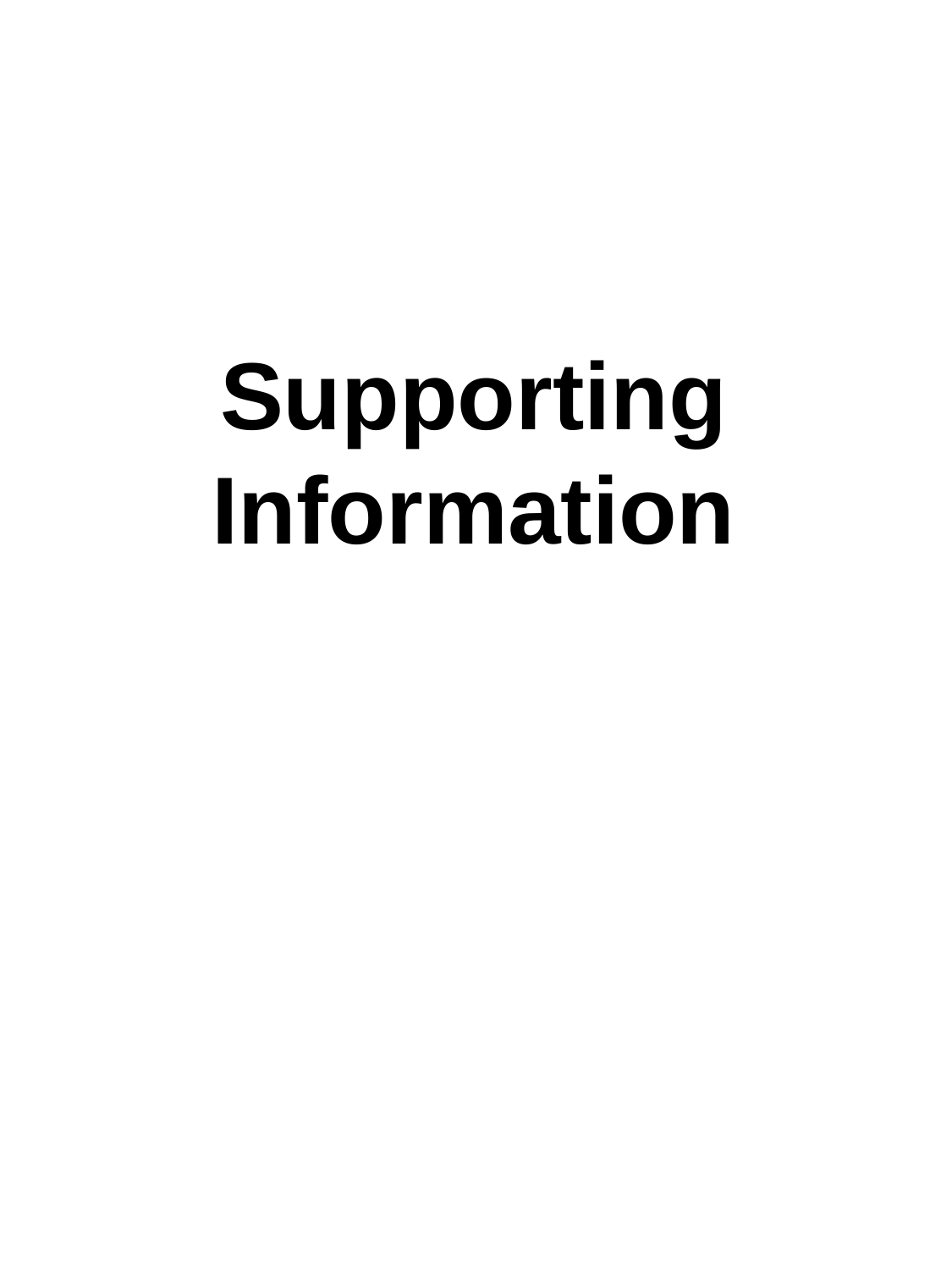

Supporting
Information

## Slide 2
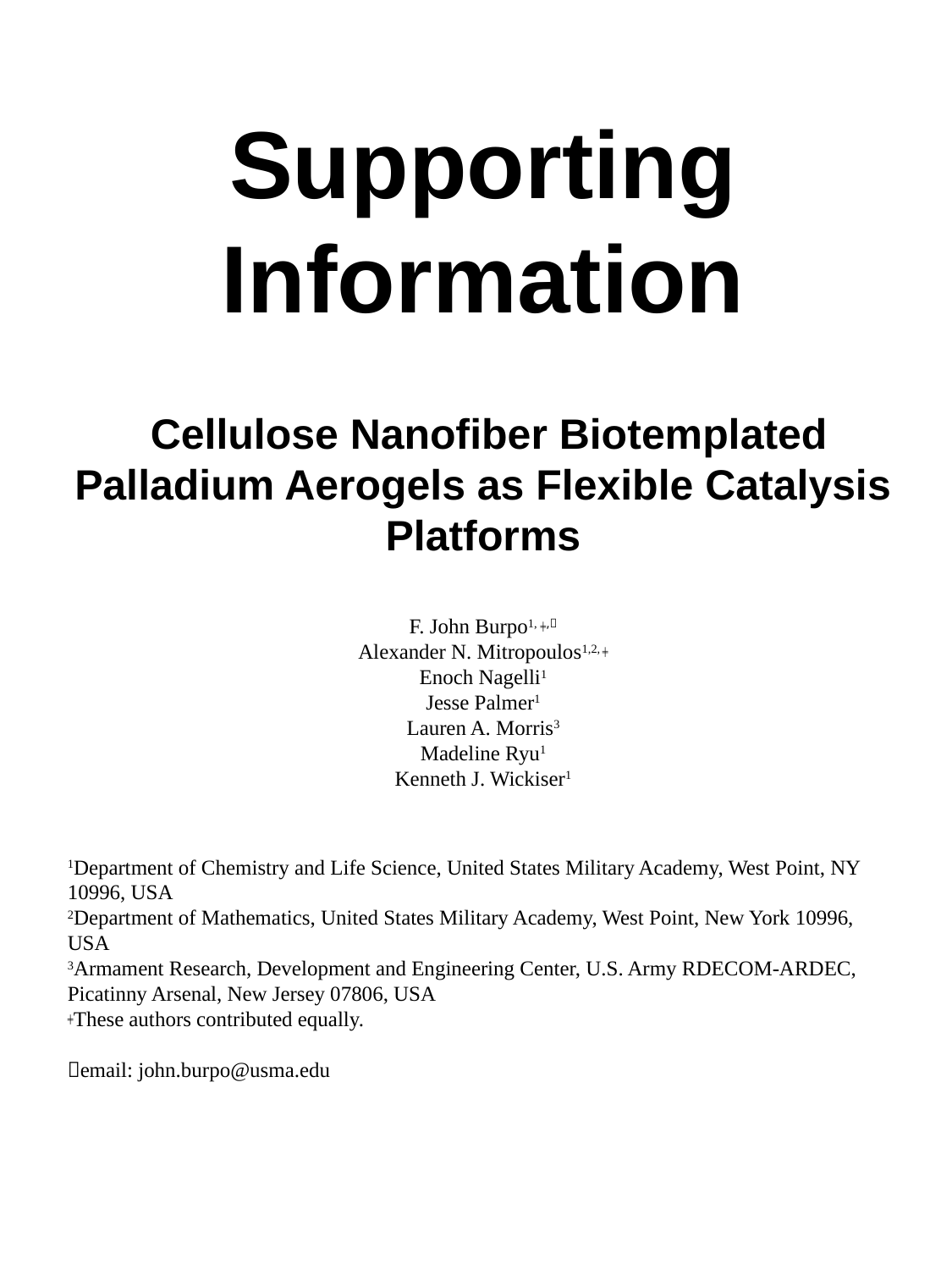

Supporting
Information
 Cellulose Nanofiber Biotemplated Palladium Aerogels as Flexible Catalysis Platforms
F. John Burpo1, ╪,
Alexander N. Mitropoulos1,2, ╪
Enoch Nagelli1
Jesse Palmer1
Lauren A. Morris3
Madeline Ryu1
Kenneth J. Wickiser1
1Department of Chemistry and Life Science, United States Military Academy, West Point, NY 10996, USA
2Department of Mathematics, United States Military Academy, West Point, New York 10996, USA
3Armament Research, Development and Engineering Center, U.S. Army RDECOM-ARDEC, Picatinny Arsenal, New Jersey 07806, USA
╪These authors contributed equally.
email: john.burpo@usma.edu

## Slide 3
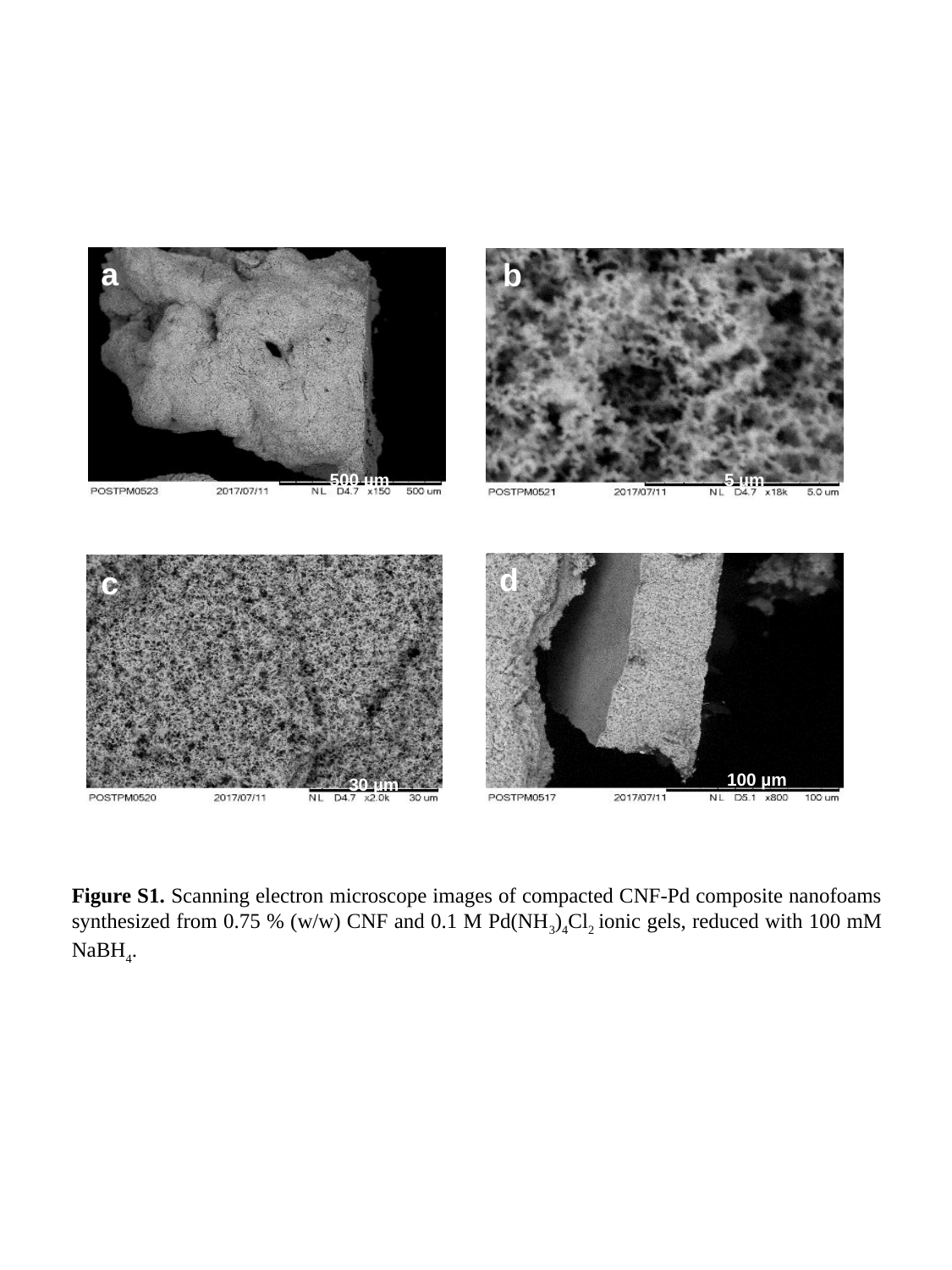

a
b
500 µm
5 µm
d
c
100 µm
30 µm
Figure S1. Scanning electron microscope images of compacted CNF-Pd composite nanofoams synthesized from 0.75 % (w/w) CNF and 0.1 M Pd(NH3)4Cl2 ionic gels, reduced with 100 mM NaBH4.

## Slide 4
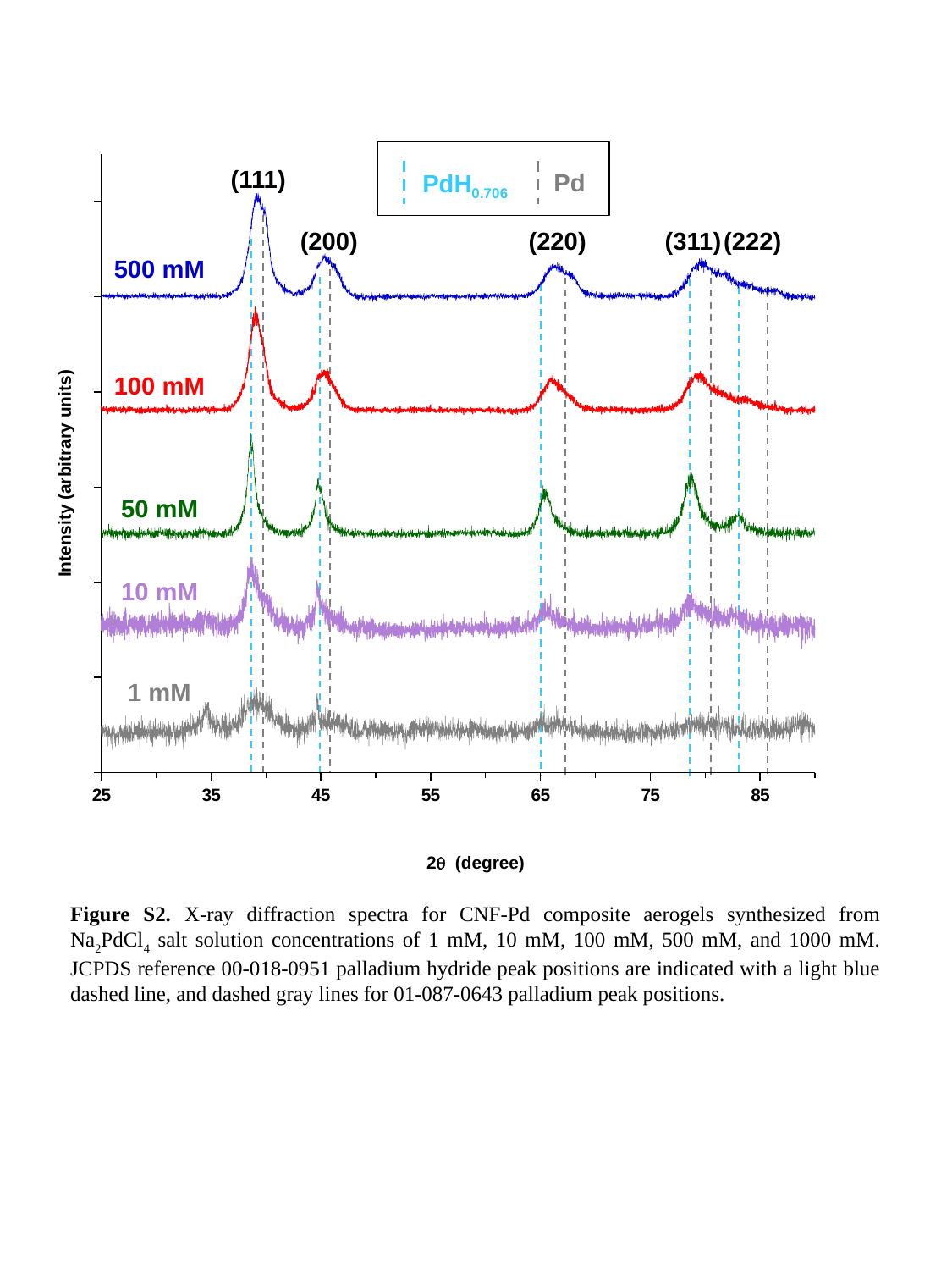

### Chart
| Category | | | | | |
|---|---|---|---|---|---|
(111)
Pd
PdH0.706
(200)
(220)
(311)
(222)
500 mM
100 mM
Intensity (arbitrary units)
50 mM
10 mM
1 mM
2 (degree)
Figure S2. X-ray diffraction spectra for CNF-Pd composite aerogels synthesized from Na2PdCl4 salt solution concentrations of 1 mM, 10 mM, 100 mM, 500 mM, and 1000 mM. JCPDS reference 00-018-0951 palladium hydride peak positions are indicated with a light blue dashed line, and dashed gray lines for 01-087-0643 palladium peak positions.

## Slide 5
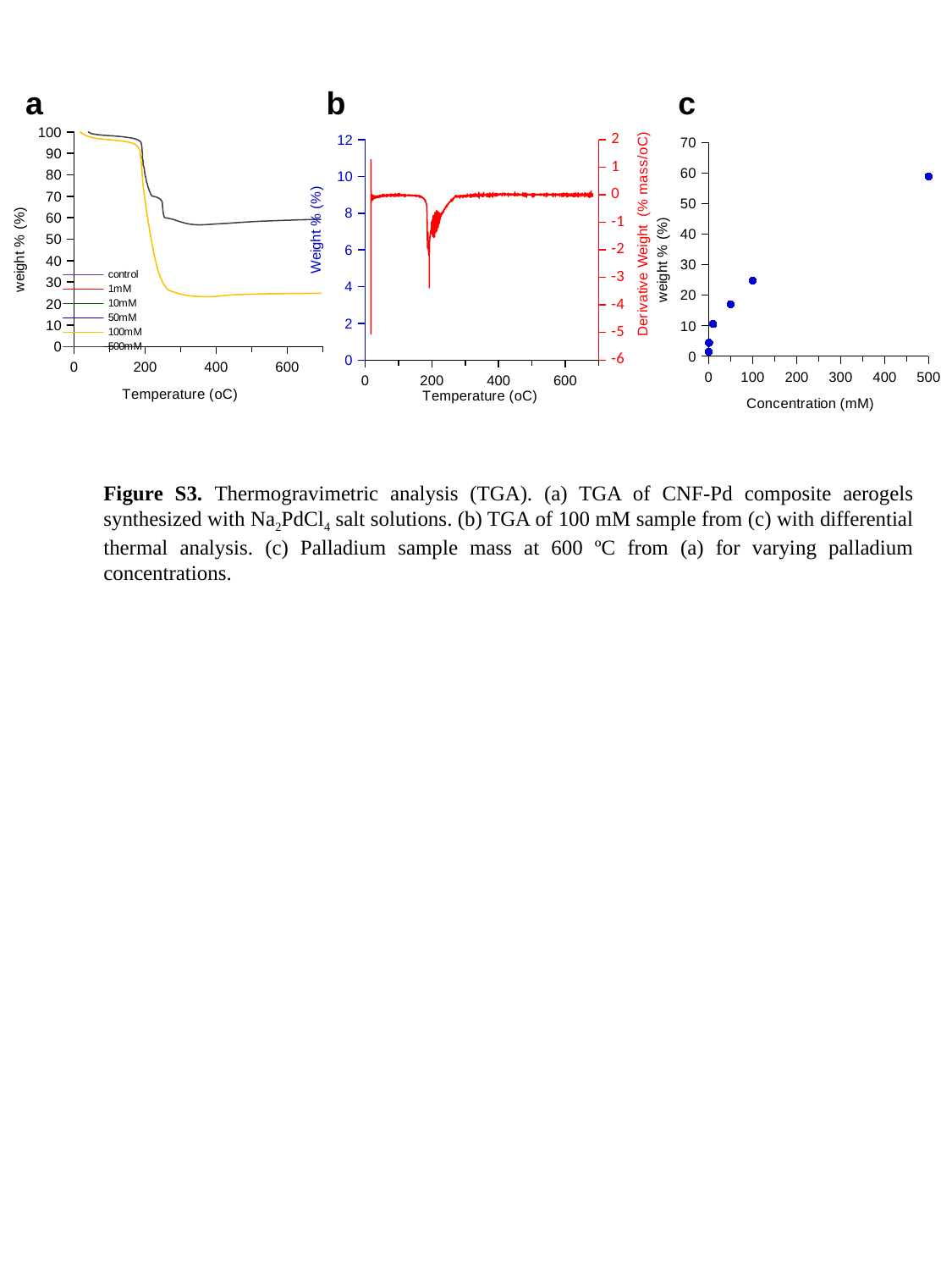

a
b
c
### Chart
| Category | control | 1mM | 10mM | 50mM | 100mM | 500mM |
|---|---|---|---|---|---|---|
### Chart
| Category | | |
|---|---|---|
### Chart
| Category | |
|---|---|Figure S3. Thermogravimetric analysis (TGA). (a) TGA of CNF-Pd composite aerogels synthesized with Na2PdCl4 salt solutions. (b) TGA of 100 mM sample from (c) with differential thermal analysis. (c) Palladium sample mass at 600 ºC from (a) for varying palladium concentrations.

## Slide 6
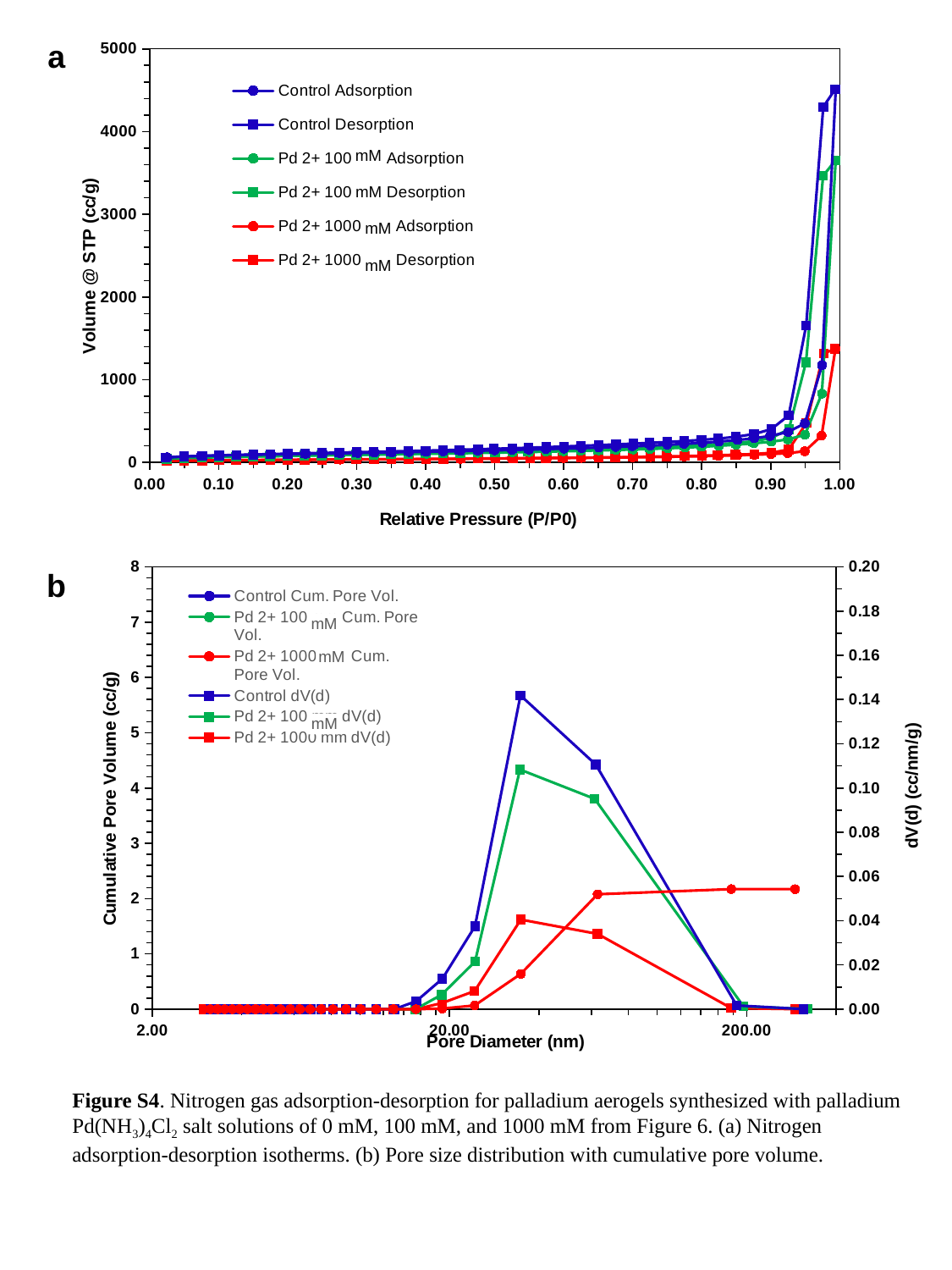

a
### Chart
| Category | | | | | | |
|---|---|---|---|---|---|---|mM
mM
mM
### Chart
| Category | | | | | | |
|---|---|---|---|---|---|---|b
mM
mM
mM
Figure S4. Nitrogen gas adsorption-desorption for palladium aerogels synthesized with palladium Pd(NH3)4Cl2 salt solutions of 0 mM, 100 mM, and 1000 mM from Figure 6. (a) Nitrogen adsorption-desorption isotherms. (b) Pore size distribution with cumulative pore volume.

## Slide 7
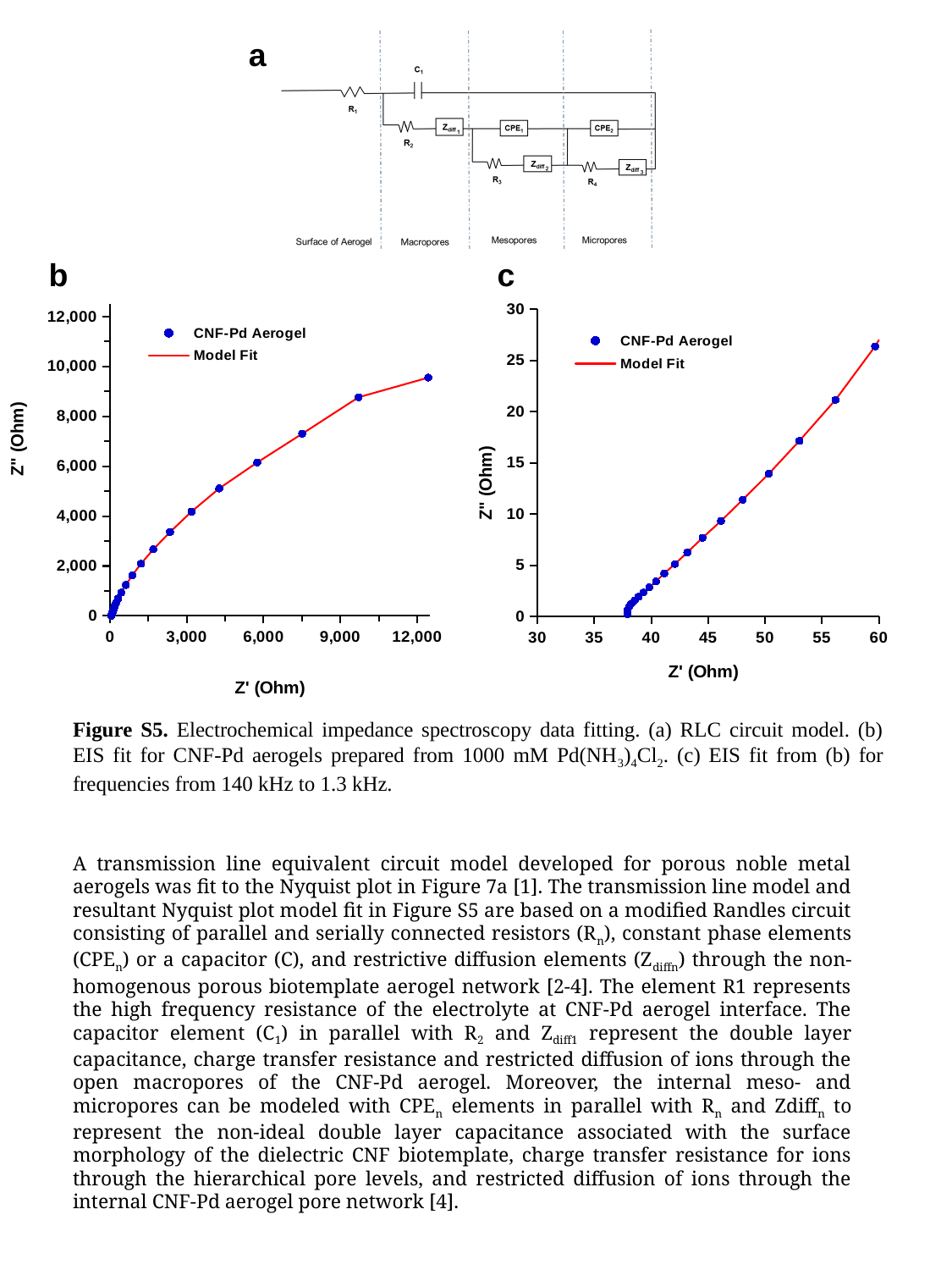

a
b
c
### Chart
| Category | | |
|---|---|---|
### Chart
| Category | | |
|---|---|---|Figure S5. Electrochemical impedance spectroscopy data fitting. (a) RLC circuit model. (b) EIS fit for CNF-Pd aerogels prepared from 1000 mM Pd(NH3)4Cl2. (c) EIS fit from (b) for frequencies from 140 kHz to 1.3 kHz.
A transmission line equivalent circuit model developed for porous noble metal aerogels was fit to the Nyquist plot in Figure 7a [1]. The transmission line model and resultant Nyquist plot model fit in Figure S5 are based on a modified Randles circuit consisting of parallel and serially connected resistors (Rn), constant phase elements (CPEn) or a capacitor (C), and restrictive diffusion elements (Zdiffn) through the non-homogenous porous biotemplate aerogel network [2-4]. The element R1 represents the high frequency resistance of the electrolyte at CNF-Pd aerogel interface. The capacitor element (C1) in parallel with R2 and Zdiff1 represent the double layer capacitance, charge transfer resistance and restricted diffusion of ions through the open macropores of the CNF-Pd aerogel. Moreover, the internal meso- and micropores can be modeled with CPEn elements in parallel with Rn and Zdiffn to represent the non-ideal double layer capacitance associated with the surface morphology of the dielectric CNF biotemplate, charge transfer resistance for ions through the hierarchical pore levels, and restricted diffusion of ions through the internal CNF-Pd aerogel pore network [4].

## Slide 8
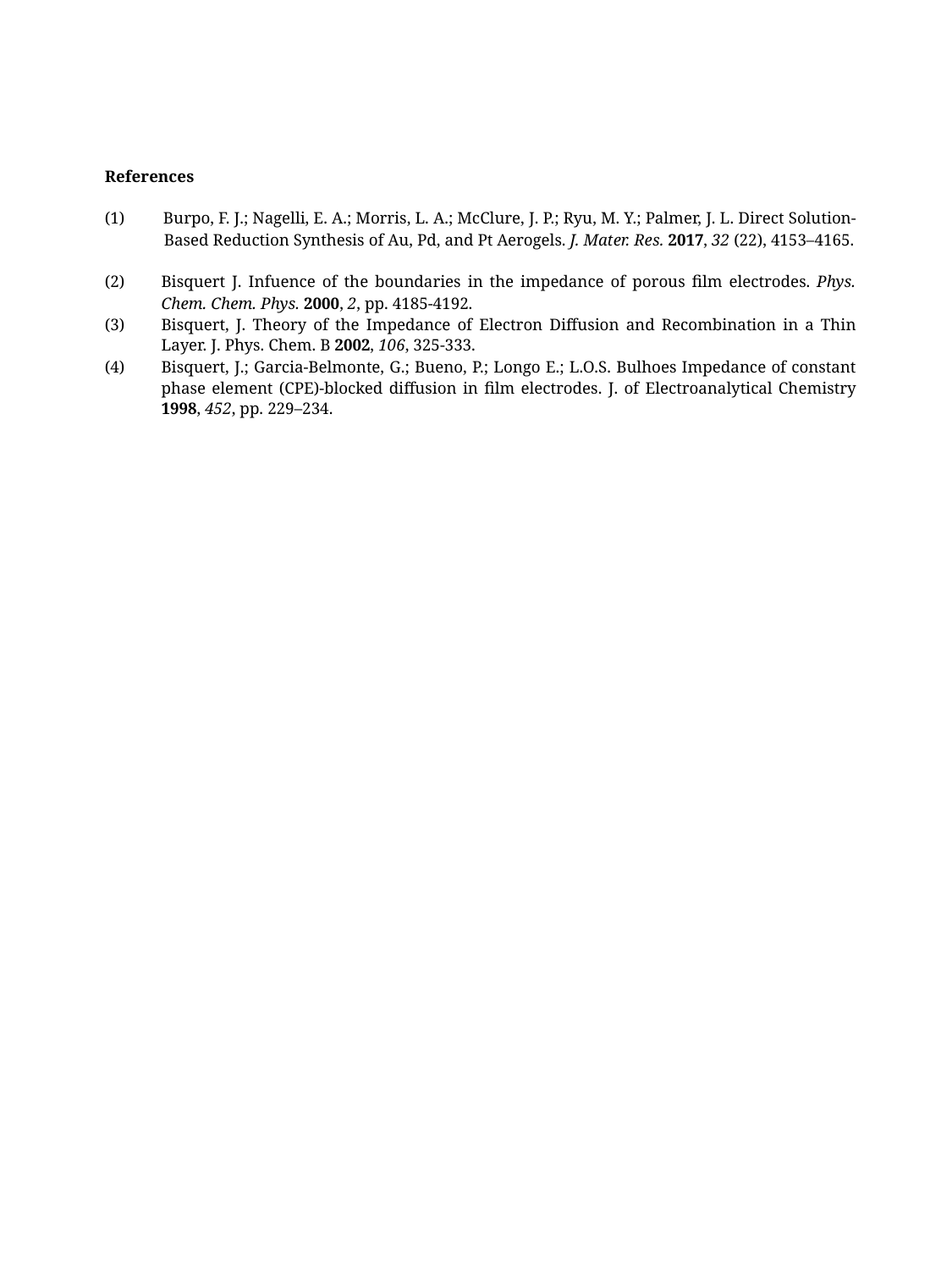

References
(1) Burpo, F. J.; Nagelli, E. A.; Morris, L. A.; McClure, J. P.; Ryu, M. Y.; Palmer, J. L. Direct Solution-
 Based Reduction Synthesis of Au, Pd, and Pt Aerogels. J. Mater. Res. 2017, 32 (22), 4153–4165.
(2)	Bisquert J. Infuence of the boundaries in the impedance of porous film electrodes. Phys. Chem. Chem. Phys. 2000, 2, pp. 4185-4192.
(3)	Bisquert, J. Theory of the Impedance of Electron Diffusion and Recombination in a Thin Layer. J. Phys. Chem. B 2002, 106, 325-333.
(4)	Bisquert, J.; Garcia-Belmonte, G.; Bueno, P.; Longo E.; L.O.S. Bulhoes Impedance of constant phase element (CPE)-blocked diffusion in film electrodes. J. of Electroanalytical Chemistry 1998, 452, pp. 229–234.
